# Markers of Dysglycaemia and Risk of Coronary Heart Disease in People without Diabetes: Reykjavik Prospective Study and Systematic Review

**DOI:** 10.1371/journal.pmed.1000278

**Published:** 2010-05-25

**Authors:** Nadeem Sarwar, Thor Aspelund, Gudny Eiriksdottir, Reeta Gobin, Sreenivasa Rao Kondapally Seshasai, Nita G. Forouhi, Gunnar Sigurdsson, John Danesh, Vilmundur Gudnason

**Affiliations:** 1 Department of Public Health and Primary Care, University of Cambridge, Cambridge, United Kingdom; 2 Icelandic Heart Association, Kopavogur, Iceland; 3 University of Iceland, Reykjavik, Iceland; 4 MRC Epidemiology Unit, Cambridge, United Kingdom; Lund University Diabetes Centre, Sweden

## Abstract

**Background:**

Associations between circulating markers of dysglycaemia and coronary heart disease (CHD) risk in people without diabetes have not been reliably characterised. We report new data from a prospective study and a systematic review to help quantify these associations.

**Methods and Findings:**

Fasting and post-load glucose levels were measured in 18,569 participants in the population-based Reykjavik study, yielding 4,664 incident CHD outcomes during 23.5 y of mean follow-up. In people with no known history of diabetes at the baseline survey, the hazard ratio (HR) for CHD, adjusted for several conventional risk factors, was 2.37 (95% CI 1.79–3.14) in individuals with fasting glucose ≥7.0 mmol/l compared to those <7 mmol/l. At fasting glucose values below 7 mmol/l, adjusted HRs were 0.95 (0.89–1.01) per 1 mmol/l higher fasting glucose and 1.03 (1.01–1.05) per 1 mmol/l higher post-load glucose. HRs for CHD risk were generally modest and nonsignificant across tenths of glucose values below 7 mmol/l. We did a meta-analysis of 26 additional relevant prospective studies identified in a systematic review of Western cohort studies that recorded fasting glucose, post-load glucose, or glycated haemoglobin (HbA_1c_) levels. In this combined analysis, in which participants with a self-reported history of diabetes and/or fasting blood glucose ≥7 mmol/l at baseline were excluded, relative risks for CHD, adjusted for several conventional risk factors, were: 1.06 (1.00–1.12) per 1 mmol/l higher fasting glucose (23 cohorts, 10,808 cases, 255,171 participants); 1.05 (1.03–1.07) per 1 mmol/l higher post-load glucose (15 cohorts, 12,652 cases, 102,382 participants); and 1.20 (1.10–1.31) per 1% higher HbA_1c_ (9 cohorts, 1639 cases, 49,099 participants).

**Conclusions:**

In the Reykjavik Study and a meta-analysis of other Western prospective studies, fasting and post-load glucose levels were modestly associated with CHD risk in people without diabetes. The meta-analysis suggested a somewhat stronger association between HbA_1c_ levels and CHD risk.

*Please see later in the article for the Editors' Summary*

## Editors' Summary

**Background:**

Among people diagnosed with type 2 diabetes mellitus (the commonest type of diabetes worldwide), poor management or lack of appropriate treatment can lead to long-term complications resulting from persistently high sugar levels in the blood. The long-term complications of type 2 diabetes are generally divided into two main groups: microvascular problems (such as nerve damage, kidney disease, and eye disorders), and macrovascular disease (such as heart disease, strokes, and peripheral vascular disease). A major goal of diabetes treatment is to keep glucose control as normal as possible through diet, weight control, exercise, and pharmacological treatments. However, it is unclear whether the link between high blood sugar and macrovascular disease (principally heart disease and strokes) also holds for people who have slightly higher than normal blood sugar levels, but in whom this level does not reach the diabetic threshold. Some previous research studies have suggested that a continuous relationship exists between blood sugar level and the risk of heart disease across the spectrum, i.e., below the diabetic threshold as well as above it. If such a relationship were confirmed this might have important implications for the management of high blood sugar levels even among people who would not normally meet the usual definition for a diagnosis of diabetes (the “diabetic threshold”).

**Why Was This Study Done?:**

Studies which examine the risk of serious, but relatively common, outcomes (such as a nonfatal heart attack or fatal heart disease), often suffer from insufficient statistical power: a large number of participants need to be recruited, and followed up over a long time, to find out whether certain factors measured at baseline (e.g., fasting glucose) are indeed associated with a particular outcome (e.g., heart attack) or not during follow up. Given the inconclusive nature of some previous studies in this area, the researchers who carried out this work wanted to gather evidence from a large prospective cohort, and a reappraisal of all existing evidence, in relation to the possible link between high blood sugar and risk of heart disease in people without diabetes.

**What Did the Researchers Do and Find?:**

In this study, the researchers report results from a prospective population-based study (in which participants are followed forward in time) from Reykjavik, Iceland. In the study, men and women without history of heart disease aged between 31 and 57 in 1966 were first invited to join the cohort, and were followed forward in time using national registries that recorded deaths (and causes of death), and incidence of heart disease. A total of 8,888 male and 9,681 female participants were recruited. At baseline, laboratory measurements were taken to record blood sugar levels using two different methods: fasting blood glucose and post-load glucose. Among the group of participants, 4,664 people were recorded as having either a nonfatal heart attack or fatal heart disease, during approximately 23 years of follow-up. In addition, the researchers attempted to identify from the published medical literature previous prospective studies conducted in Western populations that had looked at the association between blood sugar levels and risk of coronary heart disease. They requested, and obtained, re-analyses of data conducted in accordance with a common protocol for most of the identified studies and then analysed these, together with the results of the Reykjavik cohort, to produce a summary estimate (meta-analysis) of the association between blood sugar levels and risk of coronary heart disease in people without diabetes.

In the Reykjavik cohort, the researchers confirmed an increased risk of coronary heart disease among individuals with blood sugar above the diabetic threshold, as compared to those below it. However, when they looked at blood sugar in people below the diabetic threshold, they found no evidence that higher levels were strongly linked with greater risk of coronary heart disease. This held for both methods of measuring blood sugar levels (fasting and post-load).

In the meta-analysis, the researchers obtained data for 27 different studies, comprising 303,961 participants and 16,982 cases of heart disease. In this meta-analysis, very small increases in risk of heart disease were found with higher levels of blood sugar, when measured using fasting blood glucose or post-load glucose. However, studies using glycated haemoglobin (a measure of average sugar levels over the past 1–3 months or so) found this measure to be associated with a somewhat higher risk of heart disease.

**What Do these Findings Mean?:**

In this prospective cohort and wider meta-analysis, the researchers did not find evidence of a strong or continuous association between blood sugar levels and risk of heart disease amongst people without diabetes. The prospective study, and analysis of other cohorts, was large, but only looked at participants of European decent, so it is not clear whether the findings will also hold for non-European groups.

**Additional Information:**

Please access these Web sites via the online version of this summary at http://dx.doi.org/10.1371/journal.pmed.1000278.

- Information is available from the US National Diabetes Information Clearinghouse about diabetes, heart disease, and stroke
- Centers for Disease Control provides information for the public and professionals about diabetes on their diabetes minisite
- Medline Plus encyclopedia has an entry about coronary heart disease

## Introduction

Diabetes is an established risk factor for coronary heart disease (CHD). There is considerable interest in whether circulating markers of glucose metabolism are associated with risk of CHD in people without diabetes. Various measures of dysglycaemia have been assessed in long-term studies of CHD, notably: fasting glucose concentration (an indicator of steady-state glucose metabolism at the time of measurement); post-load glucose concentration (an indicator of immediate response to glycaemic stress); and glycated haemoglobin (HbA_1c_, an indicator of average blood glucose concentration over the previous 1–3 mo) [1],[2]. It has been proposed that markers of dysglycaemia may be log-linearly and importantly associated with risk of subsequent vascular disease at all levels (including below the thresholds defining diabetes) [3]–[5], but available data are not conclusive. For example, the US Preventive Services Task Force recently stated that published prospective data on fasting glucose and CHD were “inconsistent” and had “serious limitations” [6].

We report new data from the population-based Reykjavik prospective study on associations of fasting and post-load glucose levels with CHD incidence across the range of glucose values. We also did a systematic review and meta-analysis of tabular data from 26 additional relevant Western cohorts [7]–[31]. In total, the current report considers data on 303,961 participants, including 16,982 incident CHD cases.

## Methods

### Participants in the Reykjavik Study

The Reykjavik study has been described in detail previously [32]. Men born during 1907–1934 and women born during 1908–1935 who were resident in Reykjavik, Iceland and its adjacent communities on 1 December 1966 were identified in the national population register and invited to participate during five stages of recruitment between 1967 and 1991. A total of 8,888 male and 9,681 female participants without a history of myocardial infarction agreed to take part (72% response rate). Nurses administered questionnaires, made physical measurements, recorded an electrocardiogram, and collected fasting blood samples (taken after ≥8 h of fasting) at baseline. All participants have been monitored subsequently by central registries for occurrence of major cardiovascular morbidity (based on WHO MONICA [Multinational Monitoring of Trends and Determinants in Cardiovascular Disease] or similar criteria) or cause-specific mortality (based on a death certificate with International Classification of Diseases [ICD] 9 codes 410–414), with a loss to follow-up of only about 0.6% to date. During mean follow-up of 23.5 y, nonfatal MI or fatal CHD was recorded in a total of 4,664 participants, of whom 4,490 (including 3,088 men and 1,402 women) had no history of diabetes at baseline. Data were not available on incidence of stroke, diabetes, or microvascular disease. The National Bioethics Committee and the Data Protection Authority of Iceland approved the study protocol, and participants gave informed consent.

### Laboratory Methods

Fasting glucose measurement was carried out in fresh capillary whole blood within hours of blood sampling at the initial examination by a micro method on a Technicon Autoanalyzer using a modification of the W. S. Hoffman method (coefficient of variation 4%) and standardised to the Hyland Normal Clinical Chemistry Control Serum (Nygaard A/S, Oslo) [33]. Post-load glucose levels were measured 60 and 90 min after ingestion of a 50 g glucose load. Lipid and other measurements involved standard assays, as previously described [32]. HbA_1c_ measurements were not done.

### Statistical Methods

The shapes of associations with CHD risk in the Reykjavik study were characterized by calculation of hazard ratios (HRs) across tenths of glucose values, including the full range of glucose values observed. Cox proportional hazards regression models were adjusted for age, sex, smoking status, systolic blood pressure, total cholesterol, and body mass index (BMI). Ninety-five percent confidence intervals (95% CIs) were estimated from the variances that reflect the amount of information underlying each group (including the reference group) [34]. Subsidiary analyses corrected for regression dilution using serial glucose measurements made in 370 of the participants (mean interval: 12 y) [35]. In 18,333 participants without evidence of diabetes at baseline (i.e., no self-reported history and fasting blood glucose <7 mmol/l), HRs for CHD were calculated per 1 mmol/l higher glucose concentration. Impaired fasting glucose was defined using published guidelines [36]. Effect-modification was investigated by formal tests of interaction.

### Systematic Review

Three of the current investigators (NS, RG, and SRKS) sought prospective studies published between January 1970 and September 2009 that had reported on associations of fasting blood glucose, post-load glucose, and/or HbA_1c_ with incident CHD. Details of the search strategies and a flow diagram are provided in Text S1. Published studies were identified through electronic searches not limited to the English language (using MEDLINE, EMBASE, BIOSIS, and the Science Citation Index), by scanning reference lists of articles identified for all relevant studies (including review articles), and by hand searching of selected general medical journals (i.e., *BMJ, JAMA, Lancet, NEJM, PLoS Medicine, Annals of Internal Medicine, Archives of Internal Medicine*), cardiovascular/diabetes journals (i.e., *Circulation, Diabetes, Diabetes Care, European Heart Journal, Journal of the American College of Cardiology*), and epidemiological journals (i.e., *American Journal of Epidemiology, International Journal of Epidemiology*). Studies were eligible for inclusion if they: (1) did not select participants on the basis of having pre-existing vascular disease; (2) were located in Western Europe, North America, or Australasia (a restriction to reduce potential heterogeneity due to factors related to geographical location, e.g., ethnicity; see Discussion); (3) had more than 1 y of follow-up; and (4) reported on nonfatal MI (as defined by WHO MONICA or equivalent criteria, i.e., involving diagnosis based on clinical symptoms, electrocardiographic abnormalities, and/or cardiac biomarkers) and/or fatal CHD (defined by ICD criteria). Eligibility for inclusion of identified studies was considered by two investigators (RG and SRKS). Any disagreement was resolved by discussion and, if necessary, by the deciding vote of a third reviewer (NS).

A request for tabular data was sent to investigators of every eligible study identified. The following information was sought (excluding participants with a self-reported history of diabetes and/or fasting blood glucose ≥7 mmol/l at baseline), according to a uniform protocol: number of incident CHD outcomes recorded; relative risk (RR with 95% CI for CHD per unit higher dysglycaemia marker, initially after adjustment for age and sex only and then after additional adjustment for smoking status, systolic blood pressure, lipid concentrations, and BMI); the content of the glucose load and interval before post-load venipuncture; number of participants with resurvey measurements; interval between such measurements; and degree of long-term within-person variability of dysglycaemia markers (Table S1). Accuracy of the information supplied was cross-checked against published data. In the few instances of apparent discrepancy, resolution was achieved through consultation with study investigators.

### Meta-analysis

Summary RRs for CHD per unit higher dysglycaemia marker were calculated by pooling study-specific estimates using a random effects model. Analyses involved only within-study comparisons. As regards units of analysis used, 1 mmol/l higher fasting glucose corresponds approximately to 1-standard deviation (SD) higher levels; 1 mmol/l higher post-load glucose corresponds approximately to 2-SD higher levels; and 1% higher HbA_1c_ corresponds approximately to 1-SD higher levels. Consistency of findings was assessed by standard χ^2^ tests and the *I* ^2^ statistic [37]. Diversity at the study level was investigated by grouping studies by recorded characteristics and by meta-regression (including study size and duration of follow-up as continuous variables). Small-study effects were investigated [38].

## Results

### Reykjavik Study

**Figure 1:**
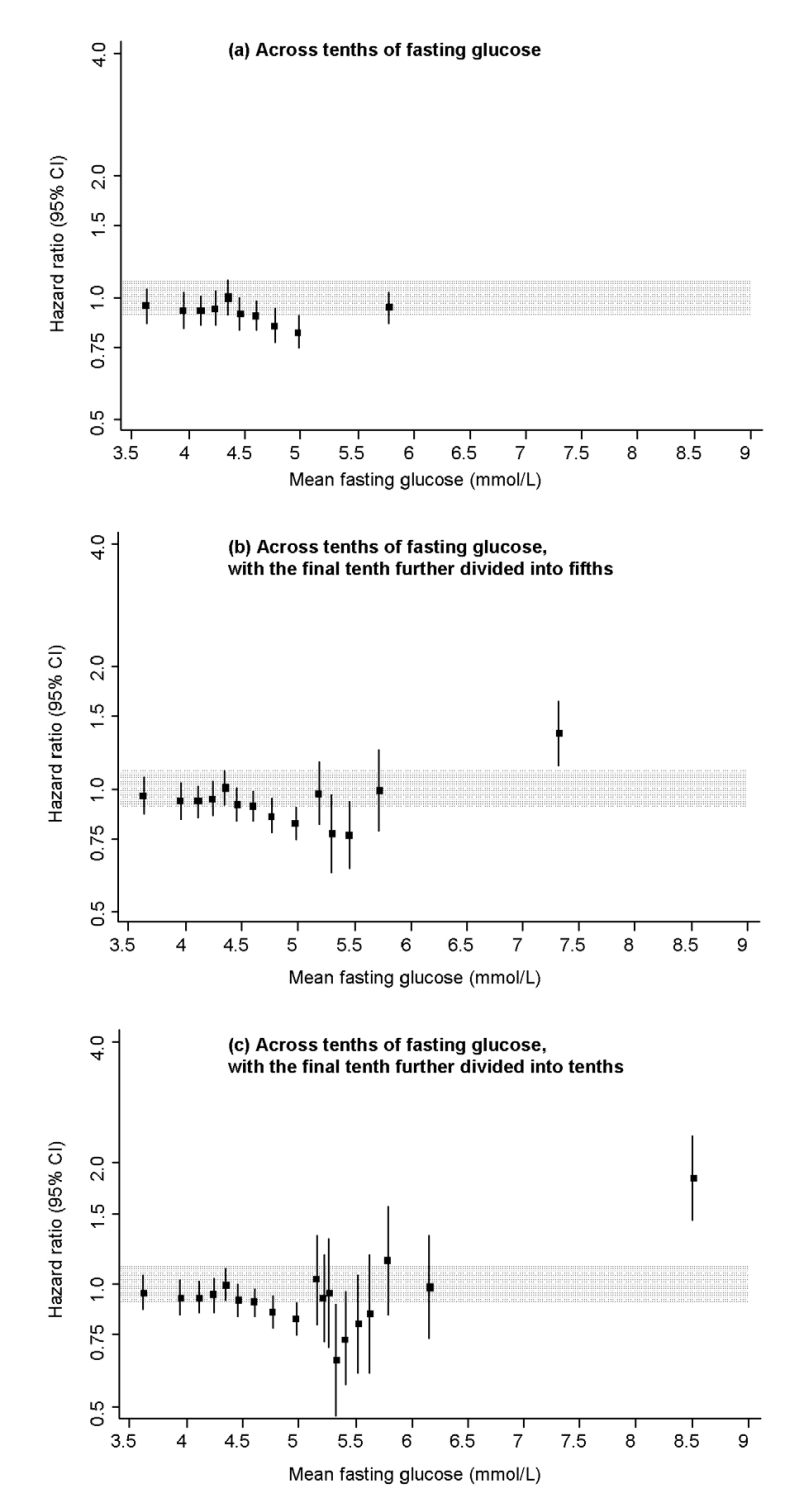
Risk of CHD across tenths of baseline fasting glucose levels in the Reykjavik Study. All hazard ratios are adjusted for age, sex, recruitment period, smoking status, systolic blood pressure, total cholesterol, and BMI, and all are compared to individuals in the middle tenth of the distribution. The grey area denotes the 95% CI of the reference group. Analyses involved the full range of glucose values (i.e., including individuals with glucose levels in the diabetic range). To limit any bias related to having a diagnosis of diabetes (e.g., medication use, lifestyle changes), however, individuals with a known history of diabetes at the baseline survey were excluded.

**Figure 2:**
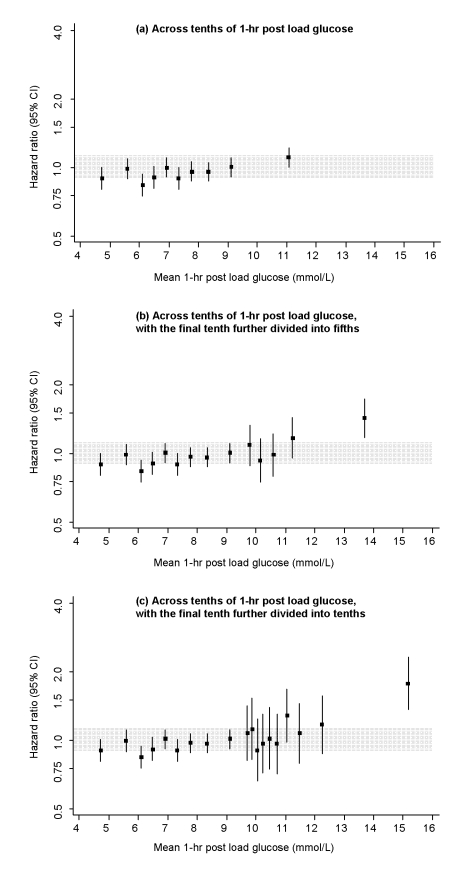
Risk of CHD across tenths of baseline 1 h post-load glucose levels in the Reykjavik Study. All hazard ratios are adjusted for age, sex, recruitment period, smoking status, systolic blood pressure, total cholesterol, and BMI, and all are compared to individuals in the middle tenth of the distribution. The grey area denotes the 95% CI of the reference group. Analyses involved the full range of glucose values (i.e., including individuals with glucose levels in the diabetic range). To limit any bias related to having a diagnosis of diabetes (e.g., medication use, lifestyle changes), however, individuals with a known history of diabetes at the baseline survey were excluded.

Baseline conventional risk factors were significantly higher in those who subsequently recorded incident CHD than in non-cases, as were fasting and 1-h post-load glucose levels (Table S2). Fasting and 1-h post-load glucose levels were each significantly correlated with several conventional risk factors and with each other (Table S3). In people without diabetes at baseline, serial measurements yielded intra-class correlation coefficients of 0.61 (95% CI 0.54–0.67) for fasting blood glucose, 0.50 (0.42–0.57) for 1-h post-load glucose, 0.59 (0.51–0.67) for total cholesterol, and 0.65 (0.54–0.77) for systolic blood pressure. In people who had no history of diabetes at baseline, HR for CHD—adjusted for age, sex, recruitment period, smoking status, systolic blood pressure, total cholesterol, and BMI (henceforth, “adjusted HR”)—was 2.37 (1.79–3.14) in those with fasting glucose ≥7.0 mmol/l compared with those <7.0 mmol/l. (HR was 1.67 [1.36–2.02] with fasting glucose ≥6.1 mmol/l compared with those <6.1 mmol/l, a definition of diabetes proposed for studies involving capillary whole blood samples [39].) In analyses across tenths of fasting glucose values, adjusted HRs for CHD were generally weak and nonsignificant at levels below 7 mmol/l (Figure 1). Findings were broadly similar for 1-h post-load glucose levels (Figure 2) and in analyses that: adjusted for age and sex only (Figure S1); corrected for regression dilution (Figure S2); assessed 90-min post-load glucose (available upon request from NS).

In people who had no history of diabetes and fasting glucose <7.0 mmol/l at baseline, adjusted HR for CHD was 0.95 (0.89–1.01) per 1 mmol/l higher fasting glucose and 1.03 (1.01–1.05) per 1 mmol/l higher 1-h post-load glucose. Similar findings were observed in analyses that: explored HRs in a range of clinically relevant subgroups (Figure S3); assessed HRs in 5-y intervals of follow-up (available upon request); or excluded people with post-load glucose levels ≥11.1 mmol/l (available upon request). Compared with individuals with fasting glucose <5.6 mmol/l, adjusted HRs for CHD were: 1.27 (0.96–1.68) with fasting glucose levels 6.1–7.0 mmol/l and 1.08 (0.87–1.33) with fasting glucose levels 5.6–6.1 mmol/l (i.e., corresponding to categories of fasting glucose concentration used to define impaired fasting glucose).

### Meta-analysis

Thirty-five potentially eligible prospective studies were identified, including the present Reykjavik study. Of these studies, 27 contributed data to the current analysis, yielding 303,961 participants, including 16,982 incident CHD cases (Table S4). This information constitutes >85% of the relevant incident CHD cases identified (Text S1 lists the noncontributing studies). Fourteen of the contributing studies were based in Western Europe, ten in North America, and three in Australia. All recruited participants from population registers or occupational settings. Fifty-six percent of the participants were male, and most were middle-aged and of European descent. For fasting glucose, there were 23 contributing studies, yielding 255,171 participants and 10,808 incident CHD cases. All studies used standard glucose assay methods and reported generally similar mean glucose values (Table S4), with four studies involving whole blood samples and 19 plasma or serum. For post-load glucose, there were 15 contributing studies, yielding 102,382 participants and 12,652 cases. Ten of these studies measured glucose 2 h after a 75 g load and five studies used other methods. For HbA_1c_, there were nine contributing studies, yielding 49,099 participants and 1,639 cases.

**Figure 3:**
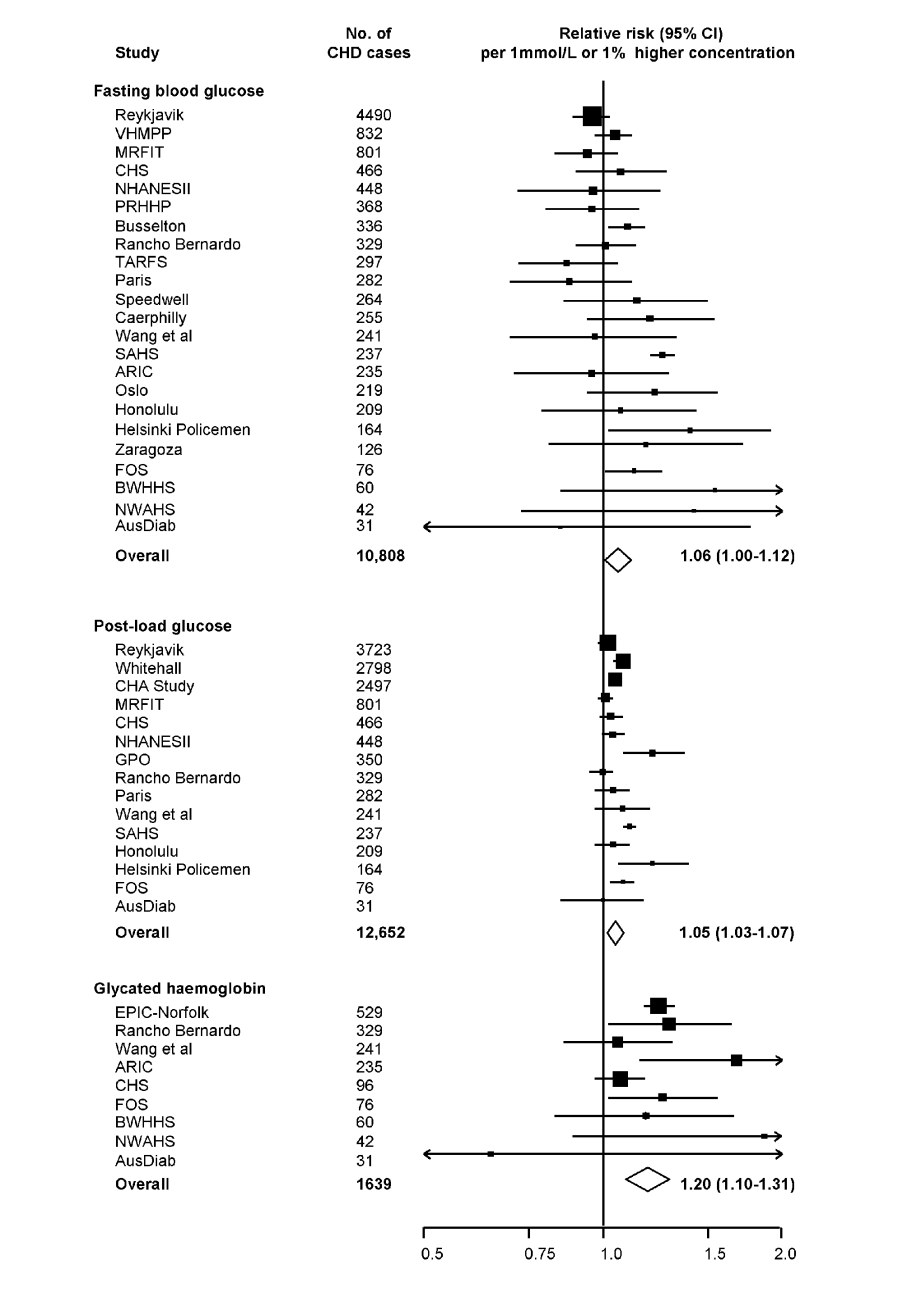
Prospective studies of markers of dysglycaemia and CHD risk in individuals without diabetes in Western populations. Analyses were restricted to individuals who did not have a self-reported history of type 2 diabetes or had a fasting blood glucose <7.0 mmol/l at baseline. Risk estimates are presented per 1 mmol/l higher fasting and post-load glucose, and per 1% higher HbA_1c_. Abbreviations as listed in Table S2.

**Figure 4:**
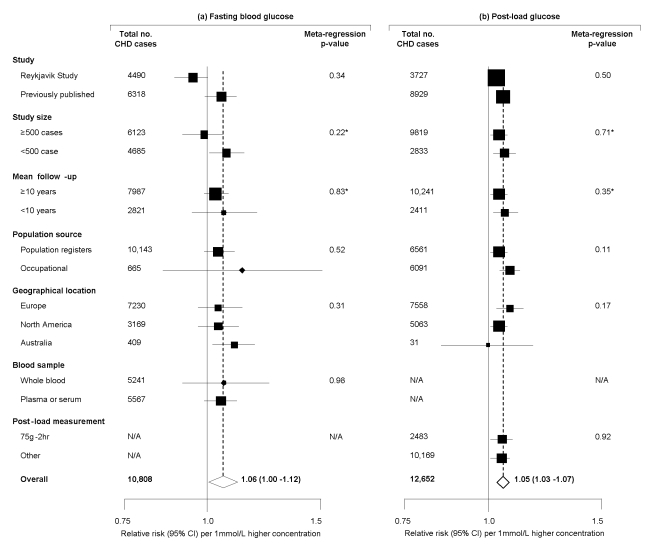
Prospective studies of fasting and post-load glucose and CHD risk in individuals without diabetes in Western population, grouped by study characteristics. Meta-regression analyses separately considered each characteristic presented. *Assessed as continuous factors.

Seven studies [8],[11],[13],[17],[23],[30] provided information on long-term serial measurements, yielding weighted intra-class correlation coefficients of 0.67 (0.66–0.69) for fasting glucose (7,834 participants, mean interval 3.5 y), 0.48 (0.46–0.50) for post-load glucose (5,617 participants, 3.5 y), and 0.69 (0.68–0.70) for HbA_1c_ (6,370 participants, 4 y). Combined RRs, adjusted for age and sex only, were: 1.12 (1.06–1.18) per 1 mmol/l higher fasting glucose; 1.08 (1.04–1.11) per 1 mmol/l higher post-load glucose; and 1.34 (1.24–1.44) per 1% higher HbA_1c_. Corresponding RRs adjusted for several conventional risk factors were: 1.06 (1.00–1.12) with fasting glucose; 1.05 (1.03–1.07) with post-load glucose; and 1.20 (1.10–1.31) with HbA_1c_ (Figure 3). There was heterogeneity in each RR (*I* ^2^ of 82% [74–88], 88% [83–93], and 42% [0–69], respectively), but little of it was explained by characteristics recorded (Figure 4). For fasting glucose, RRs were very similar in studies involving whole blood samples and in those with plasma or serum (1.05 versus 1.06). For post-load glucose, RRs were very similar in studies measuring glucose 2 h after a 75 g glucose load and in those in studies using other methods (1.05 versus 1.05). Subsidiary analyses that omitted any particular study did not materially alter the findings (e.g., omission of the Reykjavik study yielded adjusted RRs of 1.06 [1.00–1.12] with fasting glucose and 1.05 [1.03–1.08] with post-load glucose; and omission of the San Antonio Heart Study, which had unusually narrow CIs given its size, yielded adjusted RRs of 1.02 [0.99–1.06] with fasting glucose and 1.04 [1.02–1.07] with post-load glucose). There was no good evidence of small study effects (e.g., Egger test *p*>0.1 for each marker assessed). Literature-based analyses yielded broadly similar RRs with fasting glucose in the non-contributing studies to those reported here (Text S1).

## Discussion

The current data indicate that fasting and post-load glucose and HbA_1c_ each have reasonably high degrees of long-term within-person reproducibility (i.e., broadly comparable to such reproducibility values for total cholesterol and systolic blood pressure). The current meta-analysis of data from population-based Western prospective studies involving a total of >300,000 people (≈17,000 incident CHD cases) indicate that fasting glucose concentration is modestly associated with CHD risk in people without diabetes, i.e., RR for CHD was about 1.06 per 1 mmol/l higher fasting glucose. Furthermore, in the Reykjavik prospective study, RRs were generally modest and nonsignificant across glucose values below the diabetes definition (i.e., fasting glucose <7 mmol/l). We observed similar results in relation to post-load glucose. The current findings contrast with those from some smaller previous studies, which have suggested that glucose values are log-linearly and more strongly associated with CHD risk (including at glucose values lower than those defining diabetes). Because previous epidemiological estimates have influenced scientific guideline statements [40], clinical risk assessment strategies [41], burden of disease estimates [42], and public health policy recommendations [43], it may be helpful to review such efforts in the light of the current updated epidemiological evidence. Careful consideration may also need to be given to the design and interpretation of trials of CHD prevention using glucose-lowering agents in people without diabetes, as the current findings suggest that trial sample sizes required may be larger than previously anticipated [44]–[47].

In contrast with findings for glucose concentration, the current meta-analysis has indicated a RR for CHD of 1.20 per 1% higher HbA_1c_ in people without diabetes. Although RRs for CHD appear stronger with HbA_1c_ than those with glucose concentration, this possibility requires careful interpretation because: (1) the comparison is an indirect one (i.e., fasting glucose and HbA_1c_ measurements were typically not made in the same participants) and (2) fewer than one-fifth as many incident CHD cases have been reported with HbA_1c_ as with glucose concentration (so associations with HbA_1c_ cannot be quantified as reliably as those with glucose levels). Nevertheless, because the current data suggest that fasting glucose and HbA_1c_ have similar levels of within-person variability over several years, such variability seems unlikely to account for differences seen in RRs with different measures of dysglycaemia. It remains uncertain whether HbA_1c_ is a more informative measure of dysglycaemia than are fasting or post-load glucose levels, more accurately reflects processes relevant to vascular damage in response to glycation, or some combination of these possibilities [2],[48].

The strengths and potential limitations of this study merit consideration. For fasting glucose, the Reykjavik study involves more incident CHD cases than in any previous prospective study. It identified participants in population registers, achieved high response and follow-up rates, and entailed robust ascertainment of incident MI and fatal CHD. We have demonstrated the validity of the glucose measurements in capillary whole blood samples by showing: the expected strong associations of fasting glucose levels with CHD in people with values ≥7.0 mmol/l; long-term within-person consistency of glucose concentration comparable to that for systolic blood pressure and total cholesterol concentration; and similar findings as in previous studies that used plasma or serum. For post-load glucose, RRs in the Reykjavik study (involving assessment 1 h after ingestion of a 50 g glucose load) were very similar to RRs in studies involving assessment 2 h after ingestion of a 75 g glucose load.

As findings in the Reykjavik study were reinforced by a meta-analysis of tabular data from 26 other long-term prospective studies located in ten Western countries, it increases the likelihood that these results can be extrapolated, at least to other Western populations. Although our review focused only on Western cohorts to reduce heterogeneity, it may be relevant to note that the largest available prospective study in East Asia (≈3,100 incident MI outcomes) has reported similar findings to those described here, concluding that fasting glucose concentration has no clear association with MI risk below the diabetes definition [49]. Our meta-analysis included >85% of the relevant data identified by the systematic review (and a literature-based sensitivity analyses of noncontributing studies yielded broadly similar findings). Although we noted heterogeneity, it was not explained by the characteristics recorded here. Because some previous reviews did not consistently exclude people with diabetes at baseline [3],[4] or involved only fatal CHD [5], it is difficult to compare their RRs directly with the RRs observed here. A more detailed consideration of available prospective studies, perhaps on the basis of combination of individual participant data, will enable more reliable analyses under a broader range of circumstances and a more detailed investigation of potential sources of diversity.

### Conclusions

In people without diabetes, fasting and post-load glucose levels were modestly associated with CHD risk. Associations of HbA_1c_ with CHD risk in such people appeared somewhat stronger. Scientific guidelines, policies, and trial designs premised on the existence of strong, log-linear associations of fasting and post-load glucose concentration with CHD risk may benefit from review in light of these epidemiological findings.

## Supporting Information

Figure S1 Risk of coronary heart disease across tenths of baseline fasting glucose in the Reykjavik Study, adjusted for age and sex only.
(0.04 MB DOC)

Figure S2 Risk of coronary heart disease across tenths of usual fasting glucose in the Reykjavik Study.
(0.04 MB DOC)

Figure S3 Hazard ratios for coronary heart disease per 1 mmol/l higher fasting and 1-h post-load glucose concentration in individuals without diabetes in the Reykjavik Study, grouped by several characteristics.
(0.04 MB DOC)

Table S1 Copy of form used to seek tabular data for the updated meta-analysis in the present report.
(0.04 MB DOC)

Table S2 Baseline characteristics of study participants at the initial examination in the Reykjavik Study.
(0.04 MB DOC)

Table S3 Baseline correlates of fasting blood glucose and 1-h post-load glucose in participants without diabetes at the initial examination in the Reykjavik Study.
(0.04 MB DOC)

Table S4 Characteristics of prospective studies in Western populations of markers of dysglycaemia and coronary heart disease risk in individuals without diabetes included in the current analyses.
(0.10 MB DOC)

Text S1 Appendix.
(0.10 MB DOC)

## Note Added in Proof

Investigators of an additional study of fasting glucose concentration and CHD [50], involving a further 6,447 participants and 862 incident CHD cases, provided tabular data while this article was in proof. After addition of these data to the meta-analysis, the combined adjusted relative risk for CHD was 1.05 (1.00–1.10) per 1 mmol/l higher fasting glucose concentration (24 cohorts, 11,670 cases, 261,618 participants).

## Abbreviations

BMI: body mass index
CHD: coronary heart disease
CI: confidence interval
HbA_1c_: glycated haemoglobin
HR: hazard ratio
ICD: International Classification of Diseases
RR: relative risk
SD: standard deviation

## References

[1] Goldstein DE, Little RR, Lorenz RA, Malone JI, Nathan DM (2003). Tests of glycemia in diabetes.. Diabetes Care.

[2] Beckman JA, Creager MA, Libby P (2002). Diabetes and atherosclerosis: epidemiology, pathophysiology, and management.. JAMA.

[3] Coutinho M, Gerstein HC, Wang Y, Yusuf S (1999). The relationship between glucose and incident cardiovascular events. A metaregression analysis of published data from 20 studies of 95,783 individuals followed for 12.4 years.. Diabetes Care.

[4] Levitan EB, Song Y, Ford ES, Liu S (2004). Is nondiabetic hyperglycemia a risk factor for cardiovascular disease? A meta-analysis of prospective studies.. Arch Intern Med.

[5] DECODE Study Group, the European Diabetes Epidemiology Group (2001). Glucose tolerance and cardiovascular mortality: comparison of fasting and 2-hour diagnostic criteria.. Arch Intern Med.

[6] Helfand M, Buckley DI, Freeman M, Fu R, Rogers K (2009). Emerging risk factors for coronary heart disease: a summary of systematic reviews conducted for the U.S. Preventive Services Task Force.. Ann Intern Med.

[7] Ulmer H, Kelleher C, Diem G, Concin H (2004). Why Eve is not Adam: prospective follow-up in 149650 women and men of cholesterol and other risk factors related to cardiovascular and all-cause mortality.. J Womens Health (Larchmt).

[8] Eberly LE, Prineas R, Cohen JD, Vazquez G, Zhi X (2006). Metabolic syndrome: risk factor distribution and 18-year mortality in the multiple risk factor intervention trial.. Diabetes Care.

[9] Smith NL, Barzilay JI, Shaffer D, Savage PJ, Heckbert SR (2002). Fasting and 2-hour postchallenge serum glucose measures and risk of incident cardiovascular events in the elderly: the Cardiovascular Health Study.. Arch Intern Med.

[10] Saydah SH, Miret M, Sung J, Varas C, Gause D (2001). Postchallenge hyperglycemia and mortality in a national sample of U.S. adults.. Diabetes Care.

[11] Cruz-Vidal M, Garcia-Palmieri MR, Costas R, Sorlie PD, Havlik RJ (1983). Abnormal blood glucose and coronary heart disease: the Puerto Rico Heart Health Program.. Diabetes Care.

[12] Ferrie JE, Singh-Manoux A, Kivimäki M, Mindell J, Breeze E (2009). Cardiorespiratory risk factors as predictors of 40-year mortality in women and men.. Heart.

[13] Welborn TA, Wearne K (1979). Coronary heart disease incidence and cardiovascular mortality in Busselton with reference to glucose and insulin concentrations.. Diabetes Care.

[14] Scheidt-Nave C, Barrett-Connor E, Wingard DL, Cohn BA, Edelstein SL (1991). Sex differences in fasting glycemia as a risk factor for ischemic heart disease death.. Am J Epidemiol.

[15] Onat A, Hergenç G, Can G (2007). Prospective validation in identical Turkish cohort of two metabolic syndrome definitions for predicting cardiometabolic risk and selection of most appropriate definition.. Anadolu Kardiyol Derg.

[16] Balkau B, Shipley M, Jarrett RJ, Pyorala K, Pyorala M (1998). High blood glucose concentration is a risk factor for mortality in middle-aged nondiabetic men. 20-year follow-up in the Whitehall Study, the Paris Prospective Study, and the Helsinki Policemen Study.. Diabetes Care.

[17] Yarnell JW, Pickering JE, Elwood PC, Baker IA, Bainton D (1994). Does non-diabetic hyperglycemia predict future IHD? Evidence from the Caerphilly and Speedwell studies.. J Clin Epidemiol.

[18] Wang J, Ruotsalainen S, Moilanen L, Lepistö P, Laakso M (2007). The metabolic syndrome predicts cardiovascular mortality: a 13-year follow-up study in elderly non-diabetic Finns.. Eur Heart J.

[19] Stern MP, Fatehi P, Williams K, Haffner SM (2002). Predicting future cardiovascular disease: do we need the oral glucose tolerance test?. Diabetes Care.

[20] Selvin E, Coresh J, Golden SH, Brancati FL, Folsom AR (2005). Glycemic control and coronary heart disease risk in persons with and without diabetes: the atherosclerosis risk in communities study.. Arch Intern Med.

[21] Bjornholt JV, Erikssen G, Aaser E, Sandvik L, Nitter-Hauge S (1999). Fasting blood glucose: an underestimated risk factor for cardiovascular death. Results from a 22-year follow-up of healthy nondiabetic men.. Diabetes Care.

[22] Rodriguez BL, Lau N, Burchfiel CM, Abbott RD, Sharp DS (1999). Glucose intolerance and 23-year risk of coronary heart disease and total mortality: the Honolulu Heart Program.. Diabetes Care.

[23] Pyörälä M, Miettinen H, Halonen P, Laakso M, Pyörälä K (2000). Insulin resistance syndrome predicts the risk of coronary heart disease and stroke in healthy middle-aged men: the 22-year follow-up results of the Helsinki Policemen Study.. Arterioscler Thromb Vasc Biol.

[24] Marin A, Medrano MJ, Gonzalez J, Pintado H, Compaired V (2006). Risk of ischaemic heart disease and acute myocardial infarction in a Spanish population: observational prospective study in a primary-care setting.. BMC Public Health.

[25] Meigs JB, Nathan DM, D'Agostino RB, Wilson PW; Framingham Offspring Study. (2002). Fasting and postchallenge glycemia and cardiovascular disease risk: the Framingham Offspring Study.. Diabetes Care.

[26] Lawlor DA, Smith GD, Ebrahim S (2006). Does the new International Diabetes Federation definition of the metabolic syndrome predict CHD any more strongly than older definitions? Findings from the British Women's Heart and Health Study.. Diabetologia.

[27] Adams RJ, Appleton SL, Hill CL, Wilson DH, Taylor AW (2009). Independent association of HbA(1c) and incident cardiovascular disease in people without diabetes.. Obesity (Silver Spring).

[28] Barr EL, Zimmet PZ, Welborn TA, Jolley D, Magliano DJ (2007). Risk of cardiovascular and all-cause mortality in individuals with diabetes mellitus, impaired fasting glucose, and impaired glucose tolerance: the Australian Diabetes, Obesity, and Lifestyle Study (AusDiab).. Circulation.

[29] Brunner EJ, Shipley MJ, Witte DR, Fuller JH, Marmot MG. (2006). Relation between blood glucose and coronary mortality over 33 years in the Whitehall Study.. Diabetes Care.

[30] Orencia AJ, Daviglus ML, Dyer AR, Walsh M, Greenland P (1997). One-hour postload plasma glucose and risks of fatal coronary heart disease and stroke among nondiabetic men and women: the Chicago Heart Association Detection Project in Industry (CHA) Study.. J Clin Epidemiol.

[31] Khaw KT, Wareham N, Bingham S, Luben R, Welch A (2004). Association of haemoglobin A1c with cardiovascular disease and mortality in adults: the European prospective investigation into cancer in Norfolk.. Ann Intern Med.

[32] Jonsdottir LS, Sigfusson N, Gudnason V, Sigvaldason H, Thorgeirsson G (2002). Do lipids, blood pressure, diabetes, and smoking confer equal risk of myocardial infarction in women as in men? The Reykjavik Study.. J Cardiovasc Risk.

[33] Hoffman WS (1973). Rapid photoelectric method for the determination of glucose in blood and urine.. J Biol Chem.

[34] Easton DF, Peto J, Babiker AG (1991). Floating absolute risk: an alternative to relative risk in survival and case-control analysis avoiding an arbitrary reference group.. Stat Med.

[35] Clarke R, Shipley M, Lewington S, Youngman L, Collins R (1999). Underestimation of risk associations due to regression dilution in long-term follow-up of prospective studies.. Am J Epidemiol.

[36] Expert Committee on the Diagnosis and Classification of Diabetes Mellitus (2003). Follow-up report on the diagnosis of diabetes mellitus.. Diabetes Care.

[37] Higgins JP, Thompson SG, Deeks JJ, Altman DG (2003). Measuring inconsistency in meta-analyses.. BMJ.

[38] Egger M, Davey Smith G, Schneider M, Minder C (1997). Bias in meta-analysis detected by a simple, graphical test.. BMJ.

[39] WHO consultation report. Definition, diagnosis and classification of diabetes mellitus and its complications.. WHO/NCD/NCS/.

[40] Task Force on Diabetes and Cardiovascular Diseases of the European Society of Cardiology (ESC); European Association for the Study of Diabetes (EASD). (2007). Guidelines on diabetes, pre-diabetes, and cardiovascular diseases: executive summary.. Eur Heart J.

[41] Kahn R, Buse J, Ferrannini E, Stern M (2005). The metabolic syndrome: time for a critical appraisal: joint statement from the American Diabetes Association and the European Association for the Study of Diabetes.. Diabetes Care.

[42] Danaei G, Lawes CM, Vander Hoorn S, Murray CJ, Ezzati M (2006). Global and regional mortality from ischaemic heart disease and stroke attributable to higher-than-optimum blood glucose concentration: comparative risk assessment.. Lancet.

[43] Avendano M, Mackenbach JP (2006). Blood glucose levels: facing a global crisis.. Lancet.

[44] Gerstein H, Yusuf S, Riddle MC, Ryden L, Origin Trial Investigators (2008). Rationale, design, and baseline characteristics for a large international trial of cardiovascular disease prevention in people with dysglycemia: the ORIGIN Trial (Outcome Reduction with an Initial Glargine Intervention).. Am Heart J.

[45] Holman RR (2007). A new era in the secondary prevention of CVD in prediabetes - the Acarbose Cardiovascular Evaluation (ACE) trial.. Diab Vasc Dis Res.

[46] Dagenais GR, Gerstein HC, Holman R, Budaj A, DREAM Trial Investigators (2008). Effects of ramipril and rosiglitazone on cardiovascular and renal outcomes in people with impaired glucose tolerance or impaired fasting glucose: results of the Diabetes REduction Assessment with ramipril and rosiglitazone Medication (DREAM) trial.. Diabetes Care.

[47] The NAVIGATOR Study Group, Holman RR, Haffner SM, McMurray JJ, et al. (2010). Effect of Nateglinide on the Incidence of Diabetes and Cardiovascular Events.. N Engl J Med.

[48] Khaw KT, Wareham N (2006). Glycated haemoglobin as a marker of cardiovascular risk.. Curr Opin Lipidol.

[49] Sung J, Song YM, Ebrahim S, Lawlor DA (2009). Fasting blood glucose and the risk of stroke and myocardial infarction.. Circulation.

[50] Preiss D, Welsh P, Murray HM, Shepherd J, Packard C (2010). Fasting plasma glucose in non-diabetic participants and the risk for incident cardiovascular events, diabetes, and mortality: results from WOSCOPS 15-year follow-up.. http://dx.doi.org/10.1093/eurheartj/ehq095.

