# Supplementary material for: Markers of Dysglycaemia and Risk of Coronary Heart Disease in People without Diabetes: Reykjavik Prospective Study and Systematic Review
Source: PLoS Med. 2010 May 25;7(5):e1000278. doi: 10.1371/journal.pmed.1000278 (PMC2876150; doi:10.1371/journal.pmed.1000278)
Supplement: Figure S2 — Risk of coronary heart disease across tenths of usual fasting glucose in the Reykjavik Study. (0.04 MB DOC) [file pmed.1000278.s002.doc]

**Figure S2: Risk of coronary heart disease across tenths of usual* fasting glucose levels in the Reykjavik Study**

All hazard ratios are adjusted for age, sex, recruitment period, smoking status, systolic blood pressure, total cholesterol and body mass index, and all are compared to individuals in the middle tenth of the distribution. The grey area denotes the confidence interval of the reference group. Analyses involved the full range of glucose values (ie, including individuals with glucose levels in the diabetic range). To limit any bias related to having a diagnosis of diabetes (eg, medication use, lifestyle changes), however, individuals with a known history of diabetes at the baseline survey were excluded.

*Correction for regression dilution was done using a regression dilution ratio of 0.61
