# Supplementary material for: Markers of Dysglycaemia and Risk of Coronary Heart Disease in People without Diabetes: Reykjavik Prospective Study and Systematic Review
Source: PLoS Med. 2010 May 25;7(5):e1000278. doi: 10.1371/journal.pmed.1000278 (PMC2876150; doi:10.1371/journal.pmed.1000278)
Supplement: Figure S3 — Hazard ratios for coronary heart disease per 1 mmol/l higher fasting and 1-h post-load glucose concentration in individuals without diabetes in the Reykjavik Study, grouped by several characteristics. (0.04 MB DOC) [file pmed.1000278.s003.doc]

**Figure S3: Hazard ratios for coronary heart disease per 1mmol/L higher fasting and 1hr post load glucose concentration in individuals without diabetes in the Reykjavik Study, grouped by several characteristics.**

All hazard ratios were adjusted for age, sex, recruitment period, smoking status, systolic blood pressure, total cholesterol and body mass index. There was no statistical evidence for any significant interactions in the comparisons displayed in this figure (χ^2^ test for interaction P>0.01 for each). Individuals with a history of diabetes or fasting blood glucose levels ≥7.0mmol/L were excluded from these analyses.

**‡** Grouped by thirds of the distribution

✝Presented as geometric values
