# Supplementary material for: Markers of Dysglycaemia and Risk of Coronary Heart Disease in People without Diabetes: Reykjavik Prospective Study and Systematic Review
Source: PLoS Med. 2010 May 25;7(5):e1000278. doi: 10.1371/journal.pmed.1000278 (PMC2876150; doi:10.1371/journal.pmed.1000278)
Supplement: Table S1 — Copy of form used to seek tabular data for the updated meta-analysis in the present report. (0.04 MB DOC) [file pmed.1000278.s004.doc]

**Table S1: Copy of form used to seek tabular data for the updated meta-analysis in the present report**

*Please complete this table if baseline measurements of FBG, PLG and/or HbA1c were made in the study participants.* ***Please exclude all study participants with diabetes (defined as self-reported history of diabetes or fasting blood glucose ≥7.0mmol/L) from analyses.*** *If some of this information is unavailable, please provide as much detail as possible.*

| Total number of study participants who suffered either  coronary death or a non-fatal MI during follow-up | Coronary death  __ __ __ __ | non-fatal MI  __ __ __ __ |
| --- | --- | --- |
| **FASTING BLOOD GLUCOSE** |  | |
| Age and sex only adjusted relative risk (and 95% CI) for CHD*  per **1mmol/L** increase in FBG | __ . __ __ (95% CI __ . __ __ to __ . __ __) | |
| Relative risk (and 95% CI) for CHD*  per **1mmol/L** increase in FBG, adjusted for some established coronary risk factors‡ | __ . __ __ (95% CI __ . __ __ to __ . __ __) | |
| **POST LOAD GLUCOSE** |  | |
| Glucose load (grams) | __ __ . __ __ | |
| Duration between glucose load and blood collection (minutes): | __ __ __ | |
| Age and sex only adjusted relative risk (and 95% CI) for CHD*  per **1mmol/L** increase in OGTT | __ . __ __ (95% CI __ . __ __ to __ . __ __) | |
| Relative risk (and 95% CI) for CHD*  per **1mmol/L** increase in OGTT, adjusted for established coronary risk factors‡ | __ . __ __ (95% CI __ . __ __ to __ . __ __) | |
| **HbA1c** |  | |
| Age and sex only adjusted relative risk (and 95% CI) for CHD*  per **1%** increase in HbA1c | __ . __ __ (95% CI __ . __ __ to __ . __ __) | |
| Relative risk (and 95% CI) for CHD*  per **1% increase** in HbA1c, adjusted for some established coronary risk factors‡ | __ . __ __ (95% CI __ . __ __ to __ . __ __) | |

CHD defined as either coronary death or non-fatal MI.

‡ Please tick the factors that were adjusted for (or matched in the design) in the fully adjusted model:

Cigarette Smoking Blood Pressure Body Mass Index Alcohol Consumption

Total Cholesterol LDL Cholesterol HDL Cholesterol Triglycerides

Other: please specify below:

_______________________________________________________________

**Repeatability of measurements**

Either for your “controls” in this study or for some other such population, have you ever studied the **repeatability** of FBG or PLG measurements in blood samples taken some months or years apart from the same individuals? If so:

What was the correlation coefficient between such pairs of measurements? FBG 0 . __ __ PLG 0 . __ __ HbA1c 0 . __ __

How many pairs of measurements were involved in this correlation coefficient? __ __ __ __

Pairs of measurements were taken on average __ __ . __ __ months / years* apart (* please delete as appropriate)
