# Supplementary material for: Markers of Dysglycaemia and Risk of Coronary Heart Disease in People without Diabetes: Reykjavik Prospective Study and Systematic Review
Source: PLoS Med. 2010 May 25;7(5):e1000278. doi: 10.1371/journal.pmed.1000278 (PMC2876150; doi:10.1371/journal.pmed.1000278)
Supplement: Table S2 — Baseline characteristics of study participants at the initial examination in the Reykjavik Study. (0.04 MB DOC) [file pmed.1000278.s005.doc]

**Table S2: Baseline characteristics of study participants at the initial examination in the Reykjavik Study**

| **Characteristic** | **CHD cases**  **n=4490** | **Non-cases**  **n=13,843** | **P value*** |
| --- | --- | --- | --- |
|  |  |  |  |
| Age (years) | 53.9 (8.8) | 52.5 (8.8) | N/A |
| Male (%) | 3197 (69) | 5845 (42) | N/A |
| Fasting glucose (mmol/L) | 4.58 (0.96) | 4.45 (0.72) | <0.001 |
| 1-hr post load glucose (mmol/L)^‡^ | 7.70 (2.20) | 7.26 (1.85) | <0.001 |
| Current cigarette smoker (%) | 2559 (55) | 6155 (44) | <0.001 |
| Systolic blood pressure (mmHg) | 144 (23) | 137 (21) | <0.001 |
| Diastolic blood pressure (mmHg) | 89 (12) | 86 (11) | <0.001 |
| Body mass index (BMI) (kg/m^2^) | 26.0 (3.8) | 25.3 (3.9) | <0.001 |
| Total serum cholesterol (mmol/L) | 6.79 (1.16) | 6.40 (1.15) | <0.001 |
| Triglycerides** (mmol/L) | 1.14 (0.86-1.56) | 0.95 (0.72-1.30) | <0.001 |

*Adjusted for age, sex and period of recruitment

^‡^ 3723 cases and 12,385 non-cases had information on post load glucose values

**median (Inter-quartile range)
