# Supplementary material for: Markers of Dysglycaemia and Risk of Coronary Heart Disease in People without Diabetes: Reykjavik Prospective Study and Systematic Review
Source: PLoS Med. 2010 May 25;7(5):e1000278. doi: 10.1371/journal.pmed.1000278 (PMC2876150; doi:10.1371/journal.pmed.1000278)
Supplement: Table S3 — Baseline correlates of fasting blood glucose and 1-h post-load glucose in participants without diabetes at the initial examination in the Reykjavik Study. (0.04 MB DOC) [file pmed.1000278.s006.doc]

**Table S3: Baseline correlates of fasting blood glucose and 1-hr post load glucose in participants without diabetes at the initial examination in the Reykjavik Study**

|  | **Fasting blood glucose** | | | **1hr-post load glucose** | | |
| --- | --- | --- | --- | --- | --- | --- |
|  |  | | |  | | |
|  | Pearson correlation coefficient  (95% confidence interval) | t value  (adjusted for age, sex and period) | t value  (further adjusted†) | Pearson correlation coefficient  (95% confidence interval) | t value  (adjusted for age, sex and period) | t value  (further adjusted†) |
|  |  |  |  |  |  |  |
| Age | 0.06 (0.04-0.07) | - | - | 0.17 (0.15-0.18) | - | - |
| Male sex | -0.19 (-0.20 - -0.17) | - | - | -0.13 (-0.15 - -0.12) | - | - |
| Fasting blood glucose | - | - | - | 0.42 (0.41-0.44) | 55.92*** | 52.72*** |
| 1hr-post load glucose | 0.42 (0.41-0.44) | 55.92*** | 52.72*** | - | - | - |
| 90min-post load glucose | 0.31 (0.30-0.33) | 47.10*** | 43.81*** | 0.69 (0.68-0.70) | 121.19*** | 120.01*** |
| Current smokers | 0.01 (0.00-0.03) | -2.70** | 0.24 | 0.01 (0.00-0.03) | 0.27 | 2.81* |
| Systolic blood pressure | 0.21 (0.20-0.23) | 25.58*** | 25.11*** | 0.21 (0.20-0.23) | 21.21*** | 21.05*** |
| Diastolic blood pressure | 0.19 (0.17-0.20) | 19.93*** | 19.38*** | 0.18 (0.16-0.19) | 18.49*** | 18.33*** |
| Body Mass Index | 0.22 (0.21-0.24) | 28.45*** | 23.20*** | 0.15 (0.13-0.17) | 15.79*** | 11.34*** |
| Total cholesterol | 0.02 (0.01-0.04) | 4.08*** | 1.50 | 0.04 (0.03-0.06) | 3.92*** | 0.53 |
| Log_e_ triglycerides | 0.17 (0.16-0.18) | 17.26*** | 14.33*** | 0.20 (0.18-0.21) | 18.92*** | 16.43*** |

Individuals with a self-reported history of diabetes or fasting blood glucose ≥7.0mmol/L were excluded from these analyses.

T values were derived from regression of fasting blood glucose and 1-hr post load glucose on each characteristic separately. For male sex and current smokers, the Z value is reported

† Adjusted for age, sex, smoking status (current, former, never), systolic blood pressure and total cholesterol, except analysis of diastolic blood pressure was not adjusted for systolic blood pressure.

*p<0.05; **p<0.01; ***p<0.001
