# Supplementary material for: Markers of Dysglycaemia and Risk of Coronary Heart Disease in People without Diabetes: Reykjavik Prospective Study and Systematic Review
Source: PLoS Med. 2010 May 25;7(5):e1000278. doi: 10.1371/journal.pmed.1000278 (PMC2876150; doi:10.1371/journal.pmed.1000278)
Supplement: Table S4 — Characteristics of prospective studies in Western populations of markers of dysglycaemia and coronary heart disease risk in individuals without diabetes included in the current analyses. (0.10 MB DOC) [file pmed.1000278.s007.doc]

**Table S4: Characteristics of prospective studies in Western populations of markers of dysglycaemia and coronary heart disease risk in individuals without diabetes included in the current analyses**

| **Study** | **Location** | **Population source /**  **Sampling Method** | **Time of Baseline** | **No. CHD cases** | **Total**  **participants** | **Age**  **Range (yr)** | **Male**  **(%)** | **Mean follow-up**  **(years)** | **Mean (SD) baseline fasting glucose level**  **(mmol/L)** | **Assay** | | |
| --- | --- | --- | --- | --- | --- | --- | --- | --- | --- | --- | --- | --- |
|  |  |  |  |  |  |  |  |  |  | **Type of blood sample** | **Assay source** | **Assay method** |
|  |  |  |  |  |  |  |  |  |  |  |  |  |
| Reykjavik | Iceland | Population register/Complete | 1967-91 | 4490 | 18,333 | 33-81 | 48 | 23.5 | 4.5 (0.7) | Whole blood | Technicon | Hoffman |
| Whitehall^29*^ | UK | Workforce/Complete | 1967-70 | 2798 | 10,025 | 44-55 | 100 | 20 | NA | Not relevant | Technicon | Ferricyanide |
| CHA Study^30*^ | USA | Workforce/Complete | 1967-73 | 2497 | 26,745 | 18-74 | 73 | 31 | NA | Not relevant | Auto-analyzer | Hoffman |
| VHM&MP^7^ | Austria | Population register/Complete | 1985-99 | 832 | 143,478 | 20-95 | 45 | 15 | 4.8 (1.2) | Plasma | NS | Enzymatic |
| MRFIT^8^ | USA | Clinical centres/Complete | 1980-82 | 801 | 10,950 | 35-57 | 100 | 18.5 | 5.4 (0.6) | Serum | NS | NS |
| EPIC-Norfolk^31**^ | UK | GP register/Complete | 1995-97 | 529 | 10,232 | 40-79 | 45 | 6 | NA | Not relevant | BioRad | HPLC |
| CHS^9^ | USA | Population register/Random | 1989-93 | 466 | 3260 | >65 | 37 | 8.5 | 5.4 (0.5) | Serum | Eastman Kodak | NS |
| NHANESII^10^ | USA | Population register/Random | 1976-80 | 448 | 3092 | 30-74 | 45 | 13 | 5.1 (NS) | Plasma | NS | NS |
| PRHHP^11^ | USA | Population register/Complete | 1965 | 368 | 8624 | 45-64 | 100 | 12 | 5.0 (0.6) | Whole blood | NS | NS |
| GPO Study^12^ | UK | Workforce/Complete | 1966 | 350 | 1788 | 35-70 | 66 | 40 | NA | Not relevant | Technicon | Ferricyanide |
| Busselton^13^ | Australia | Electoral Roll/Complete | 1981 | 336 | 1612 | 40-89 | 45 | 15 | 4.9 (0.6) | Plasma | NS | NS |
| Rancho Bernardo^14^ | USA | Population register/Random | 1972-74 | 329 | 3458 | 40-79 | 43 | 14 | 5.7 (NS) | Plasma | In house | Hexokinase |
| TARFS^15^ | Turkey | Population register/Random | 1997/98 | 297 | 2553 | ≥28 | 49 | 7.5 | 5.2(NS) | Plasma | NS | NS |
| Paris Prospective^16^ | France | Workforce/Complete | 1968-74 | 282 | 6629 | 43-52 | 100 | 20 | 5.6 (0.6) | Plasma | Technicon | Ferricyanide |
| Speedwell^17^ | UK | GP lists/Complete | 1979-83 | 264 | 2186 | 45-63 | 100 | 13 | 4.9 (NS)‡ | Plasma | Beckman | Oxygen rate |
| Caerphilly^17^ | UK | Population register/Complete | 1979-83 | 255 | 2250 | 45-59 | 100 | 13 | 4.9 (NS)‡ | Plasma | Beckman | Oxygen rate |
| Wang et al.^18^ | Finland | Population register/Random | 1986-88 | 241 | 937 | 65-74 | 37 | 13.5 | NS | Plasma | NS | NS |
| SAHS^19^ | USA | Population register/Random | 1979-88 | 237 | 3902 | 25-64 | 40 | 7.5 | NS | Plasma | Abbott | NS |
| ARIC^20^ | USA | Population register/Complete | 1990-92 | 235 | 14,166 | 45-64 | NS | 8 | 5.5 (NS) | Serum | NS | Hexokinase |
| Oslo Study^21^ | Norway | Workforce/Complete | 1972 | 219 | 1973 | 40-59 | 100 | 22 | 4.3 (NS)⁪ | Whole blood | In house | Oxidase |
| Honolulu^22^ | USA | Population register/Complete | 1991-93 | 209 | 2322 | 71-93 | 100 | 6 | NS | Serum | Auto-analyzer | Hoffman |
| Helsinki Policemen^23^ | Finland | Workforce/Complete | 1971-72 | 164 | 970 | 34-64 | 100 | 22 | 4.9 (0.5) | Whole blood | NS | O-toluidine |
| Zaragoza^24^ | Spain | Primary care centres/Complete | 1994 | 126 | 5630 | >25 | 45 | 5 | NS | Serum | Gluco-quant | Hexokinase |
| FOS^25^ | USA | Population register/Complete | 1991-95 | 76 | 3370 | 54† | 46 | 4 | 5.4 (NS) | Plasma | Abbott | Hexokinase |
| BWHHS^26^ | UK | Population register/Random | 1999-01 | 60 | 3589 | 60-79 | 0 | 4 | 5.9¶ (NS) | Plasma | Hitachi | Oxidase |
| NWAHS^27^ | Australia | Household register/Random | 2000 | 42 | 3061 | ≥18 | 45 | 3.5 | NS | Plasma | NS | NS |
| AusDaib^28^ | Australia | Population register/Complete | 1999-00 | 31 | 8826 | ≥25 | 45 | 4.7 | NS | Plasma | Olympus | NS |
| **Total** |  |  |  | **16,982** | **303,961** |  |  |  |  |  |  |  |

‡ Combined mean across both cohorts; ⁪ *These studies recorded only post-load glucose levels; **This study recorded only HbA_1c_ levels; † mean age; ¶ geometric mean

CHD, non-fatal myocardial infarction or coronary death; NS not stated; NA not applicable; CHA Chicago Heart Association; VHM&PP Voralberg Health Monitoring and Promotion Program; MRFIT Multiple Risk Factor Intervention Trial; CHS Cardiovascular Health Study; NHANES II Second National Health and Nutrition Examination Survey; PRHHP Puerto Rico Heart Health Program; GPO General Post Office; TARFS Turkish Adults Risk Factors Study, SAHS San Antonio Heart Study; ARIC Atherosclerosis Risk in Communities; FOS Framingham Offspring Study; BWHHS British Women’s Heart Health Study; NWAHS North West Adelaide Health Study; AusDiab Australian Diabetes, Obesity, and Lifestyle Study; NS Not stated; NA Not available; GP General practice
