# Supplementary material for: Markers of Dysglycaemia and Risk of Coronary Heart Disease in People without Diabetes: Reykjavik Prospective Study and Systematic Review
Source: PLoS Med. 2010 May 25;7(5):e1000278. doi: 10.1371/journal.pmed.1000278 (PMC2876150; doi:10.1371/journal.pmed.1000278)
Supplement: Text S1 — Appendix. (0.10 MB DOC) [file pmed.1000278.s008.doc]

**SUPPLEMENTARY TEXT S1**

**Search strategy**

Computer-based searches combined search terms detailed below:

*i) Pubmed strategy to identify studies of fasting glucose, post load glucose or glycated haemoglobin*

((((((((((((((((((glucose)) OR ((blood glucose))) OR ((plasma glucose))) OR ((serum glucose))) OR ((capillary glucose))) OR ((fasting glucose))) OR ((fasting blood glucose))) OR ((fasting plasma glucose))) OR ((fasting serum glucose))) OR ((fasting capillary glucose))) OR ((whole blood glucose))) OR ((fasting whole blood glucose)))) OR ((HbA1c OR glycosylated haemoglobin OR glycated haemoglobin))) OR ((((((((glycaemia)) OR ((glycemia))) OR ((fasting glycemia))) OR ((fasting glycaemia))) OR ((postload glycemia))) OR ((postload glycaemia))))) OR (((((blood sugar)) OR ((blood sugar levels))) OR ((high blood sugar levels))))) OR (((((((post load glucose)) OR ((post load glucose blood glucose))) OR ((post load glucose plasma glucose))) OR ((post load glucose serum glucose))) OR ((oral glucose tolerance test OR OGTT))))) OR ((((("Glucose Tolerance Test"[Mesh] OR "Glucose Intolerance"[Mesh] OR "Blood Glucose"[Mesh])) OR (("Hemoglobin A, Glycosylated"[Mesh] OR "hemoglobin A1c protein, human "[Substance Name]))) OR (("Glucose Tolerance Test"[Mesh]))))

*ii) Pubmed strategy to identify studies of coronary heart disease*

(((((((((coronary heart disease OR coronary artery disease OR ischaemic heart disease OR ischemic heart disease)) OR ((myocardial infarction OR acute myocardial infarction OR MI OR AMI OR heart attack))) OR ((non-fatal myocardial infarction OR non-fatal MI))) OR ((fatal myocardial infarction OR fatal MI))) OR ((coronary death))) OR ((acute coronary syndrome OR ACS))) OR (("Coronary Disease"[Mesh]))) OR (("Myocardial Ischemia"[Mesh]))) OR (("Coronary Artery Disease"[Mesh]))

*iii) Pubmed strategy to identify prospective studies*

((((((((((prospective study)) OR ((cohort study))) OR ((longitudinal study))) OR ((prospective cohort study))) OR ((nested case control study))) OR ((case cohort study)))) OR (("Prospective Studies"[Mesh]))) OR (("Cohort Studies"[Mesh]))) OR (("Longitudinal Studies"[Mesh]))

Parts i, ii and iii were combined using ‘AND’ to search Pubmed. Each part was specifically translated for searching alternative databases.

| **Meta-analysis of Observational Studies in Epidemiology (MOOSE) checklist^9^** | |
| --- | --- |
| **Reporting Checklist** | **Place found in current paper** |
| Reporting of background should include |  |
| Problem definition | Page 3 |
| Hypothesis statement | Page 3 |
| Description of study outcome(s) | Pages 4-5 |
| Type of exposure or intervention used | Pages 4-5 |
| Type of study designs used | Pages 4-5 |
| Study population | Pages 4-5 |
| Reporting of search strategy should include |  |
| Qualifications of searchers (eg, librarians and investigators) | Page 4 |
| Search strategy, including time period included in the synthesis and keywords | Appendix |
| Effort to include all available studies, including contact with authors | Page 5 and Appendix |
| Databases and registries searched | Appendix |
| Search software used, name and version, including special features used (eg, explosion) | Appendix |
| Use of hand searching (eg, reference lists of obtained articles) | Appendix |
| List of citations located and those excluded, including justification | Appendix |
| Method of addressing articles published in languages other than English | Appendix |
| Method of handling abstracts and unpublished studies | Appendix |
| Description of any contact with authors | Page 5 and Appendix |
| Reporting of methods should include |  |
| Description of relevance or appropriateness of studies assembled for assessing the hypothesis to be tested | Page 6 |
| Rationale for the selection and coding of data (eg, sound clinical principles or convenience) | Pages 4-5 |
| Documentation of how data were classified and coded (eg, multiple raters, blinding, and interrater reliability) | Page 5 |
| Assessment of confounding (eg, comparability of cases and controls in studies where appropriate) | Pages 5-7 |
| Assessment of study quality, including blinding of quality assessors; stratification or regression on possible predictors of study results | Pages 6-7, Table 2 |
| Assessment of heterogeneity | Pages 5-7, Figure 4 |
| Description of statistical methods (eg, complete description of fixed or random effects models, justification of whether the chosen models account for predictors of study results, dose-response models, or cumulative meta-analysis) in sufficient detail to be replicated | Page 5 |
| Provision of appropriate tables and graphics | Figures 3-4, Table 2, eTable 1 |
| Reporting of results should include |  |
| Graphic summarizing individual study estimates and overall estimate | Figure 3 and Appendix |
| Table giving descriptive information for each study included | Table 2 |
| Results of sensitivity testing (eg, subgroup analysis) | Figure 4 and Appendix |
| Indication of statistical uncertainty of findings | Figures 3-4 |
| Reporting of discussion should include |  |
| Quantitative assessment of bias (eg, publication bias) | Page 9 |
| Justification for exclusion (eg, exclusion of non–English-language citations) | Pages 4-5, 9 |
| Assessment of quality of included studies | Page 9 |
| Reporting of conclusions should include |  |
| Consideration of alternative explanations for observed results | Pages 8-9 |
| Generalization of the conclusions (ie, appropriate for the data presented and within the domain of the literature review) | Page 9 |
| Guidelines for future research | Page 9 |
| Disclosure of funding source | Page 10 |

**References for Supplementary Text S1**

1. Sorkin JD, Muller DC, Fleg JL, Andres R. (2005) The relation of fasting and 2-h postchallenge plasma glucose concentrations to mortality: data from the Baltimore Longitudinal Study of Aging with a critical review of the literature. Diabetes Care 28:2626-32.

2. Gao L, Matthews FE, Sargeant LA, Brayne C; MRC CFAS. (2008) An investigation of the population impact of variation in HbA1c levels in older people in England and Wales: from a population based multi-centre longitudinal study. BMC Public Health 8:54.

3. DECODE Study Group, the European Diabetes Epidemiology Group. (2001) Glucose tolerance and cardiovascular mortality: comparison of fasting and 2-hour diagnostic criteria. Arch Intern Med 161:397-405.

4. Meisinger C, Wölke G, Brasche S, Strube G, Heinrich J. (2006) Postload plasma glucose and 30-year mortality among nondiabetic middle-aged men from the general population: the ERFORT Study. Ann Epidemiol 16:534-9.

5. Pradhan AD, Rifai N, Buring JE, Ridker PM. (2007) Hemoglobin A1c predicts diabetes but not cardiovascular disease in nondiabetic women. Am J Med 120:720-7.

6. Simons LA, Simons J, Friedlander Y, McCallum J. (2008) Usefulness of fasting plasma glucose to predict mortality or coronary heart disease in persons > or = 60 years of age without diabetes mellitus or in those with undiagnosed diabetes mellitus (from The Dubbo Study). Am J Cardiol 102:831-4.

7. Tomas-Abadal L, Varas-Lorenzo C, Bernades-Bernat E, Balaguer-Vintro I. (1994) Coronary risk factors and a 20-year incidence of coronary heart disease and mortality in a Mediterranean industrial population. The Manresa Study, Spain. Eur Heart J 15:1028-36.

8. Welin L, Bresäter LE, Eriksson H, Hansson PO, Welin C, et al. (2003) Insulin resistance and other risk factors for coronary heart disease in elderly men. The Study of Men Born in 1913 and 1923. Eur J Cardiovasc Prev Rehabil 10 :283-8.

9. Stroup DF, Berlin JA, Morton SC, Olkin I, Williamson GD, et al. (2000) Meta-analysis of observational studies in epidemiology: a proposal for reporting. Meta-analysis Of Observational Studies in Epidemiology (MOOSE) group. JAMA 283:2008-12.
